# Syndromic Algorithms for Detection of *Gambiense* Human African Trypanosomiasis in South Sudan

**DOI:** 10.1371/journal.pntd.0002003

**Published:** 2013-01-17

**Authors:** Jennifer J. Palmer, Elizeous I. Surur, Garang W. Goch, Mangar A. Mayen, Andreas K. Lindner, Anne Pittet, Serena Kasparian, Francesco Checchi, Christopher J. M. Whitty

**Affiliations:** 1 Faculty of Infectious and Tropical Diseases, London School of Hygiene and Tropical Medicine, London, United Kingdom; 2 Medical Emergency Relief International, Nimule, South Sudan; 3 Medical Mission Institute, Department of Tropical Medicine, Würzburg, Germany; 4 Département Médico-Chirurgical de Pédiatrie, Centre Hospitalier Universitaire Vaudois, Lausanne, Switzerland; 5 Médecins sans frontiers, Montréal, Canada; University of Ghana, Ghana

## Abstract

**Background:**

Active screening by mobile teams is considered the best method for detecting human African trypanosomiasis (HAT) caused by *Trypanosoma brucei gambiense* but the current funding context in many post-conflict countries limits this approach. As an alternative, non-specialist health care workers (HCWs) in peripheral health facilities could be trained to identify potential cases who need testing based on their symptoms. We explored the predictive value of syndromic referral algorithms to identify symptomatic cases of HAT among a treatment-seeking population in Nimule, South Sudan.

**Methodology/Principal Findings:**

Symptom data from 462 patients (27 cases) presenting for a HAT test via passive screening over a 7 month period were collected to construct and evaluate over 14,000 four item syndromic algorithms considered simple enough to be used by peripheral HCWs. For comparison, algorithms developed in other settings were also tested on our data, and a panel of expert HAT clinicians were asked to make referral decisions based on the symptom dataset. The best performing algorithms consisted of three core symptoms (sleep problems, neurological problems and weight loss), with or without a history of oedema, cervical adenopathy or proximity to livestock. They had a sensitivity of 88.9–92.6%, a negative predictive value of up to 98.8% and a positive predictive value in this context of 8.4–8.7%. In terms of sensitivity, these out-performed more complex algorithms identified in other studies, as well as the expert panel. The best-performing algorithm is predicted to identify about 9/10 treatment-seeking HAT cases, though only 1/10 patients referred would test positive.

**Conclusions/Significance:**

In the absence of regular active screening, improving referrals of HAT patients through other means is essential. Systematic use of syndromic algorithms by peripheral HCWs has the potential to increase case detection and would increase their participation in HAT programmes. The algorithms proposed here, though promising, should be validated elsewhere.

## Author Summary

Human African trypanosomiasis (HAT or sleeping sickness) is an almost always fatal disease affecting poor people in rural, conflict-affected areas of sub-Saharan Africa. It is difficult to diagnose. Effective treatment exists, but because diagnostic and treatment services are usually based only in hospitals, many HAT patients in rural areas are never detected. Control programmes aim periodically to extend testing services via mobile teams (active screening) but their expense and operational issues severely restrict their use. We explored the predictive value of different combinations of symptoms that were present in a treatment-seeking population to identify people infected with HAT. Through this approach, we identified a simple four-symptom referral algorithm that, if replicable, has the potential to identify one HAT patient for every ten patients referred through subsequent testing. It would identify most symptomatic HAT patients who seek treatment, if systematically applied by non-specialist healthcare workers already working in these areas. As these types of health workers are rarely included in formal HAT control efforts, teaching this algorithm also represents an opportunity to decentralise life-saving knowledge, and contribute to endemic populations' long-term empowerment and ability to help control this disease.

## Introduction

Found in remote sub-Saharan areas where health systems are often weak and/or destabilised by armed conflict, human African trypanosomiasis (HAT, or sleeping sickness) is one of the world's most neglected tropical diseases (NTDs). It is caused by trypanosome parasites that are transmitted primarily through the bites of infected tsetse flies (*Glossina*). It is nearly always fatal if untreated. HAT caused by *Trypanosoma brucei gambiense* represents more than 90% of global HAT burden and is endemic in small geographic foci in 24 countries of west and central Africa [1]. Humans are assumed to be the main reservoir of infection in these areas.

In the first stage of disease, when parasites are found in the blood, lymph and organ systems, HAT can be asymptomatic or involve non-specific symptoms. Patients are considered to be in the second stage of disease once there is evidence that parasites have entered the brain and cerebro-spinal fluid (CSF), and it is at this time that characteristic symptoms are more likely to appear, involving mental and physical deterioration progressing to death [2], [3]. The natural duration of *gambiense* HAT is thought to be almost a year and a half for each stage [4].

HAT diagnosis is currently not feasible in peripheral primary health care structures as it requires refrigeration, electricity, specific equipment and a high level of technical expertise [5], [6]. Systematic active screening (AS) of at-risk populations using laboratory-equipped mobile teams is used to increase access to treatment and reduce the infectious pool [1], [7], [8], [9], but is considerably more expensive than passive screening (PS) at static facilities. Despite calls for an intensification of control activities coherent with an elimination aim [10], funding for such activities on the ground, especially for AS and in the most endemic countries, has recently become worryingly scarce [11], [12]. In South Sudan only 445 people were actively screened for infection in 2010 [13]. In this resource context, it would be useful to develop case-detection and treatment strategies that can be sustainably implemented by national and local control programmes. Furthermore, targeting testing to symptomatic patients, who probably have a higher probability of being HAT cases than the general population, may be more cost-effective and would reduce the risk of drug related adverse events among false positives [14].

One option is to involve non-specialist healthcare workers (HCWs) in peripheral, first and second-tier primary healthcare (PHC) facilities in syndromic recognition of suspect cases; these HCWs are a resource that are often over-looked in vertical HAT control programmes [15], [16]. The WHO cautions against the exclusive use of signs and symptoms for HAT diagnosis since these are known to be nonspecific in HAT and their frequency varies widely between individuals and potentially even geographic regions [17]. However, a simple syndromic algorithm, if sufficiently sensitive, could empower HCWs to recognise and refer suspect HAT patients to a specialised PS facility for diagnostic HAT testing, and could therefore be a useful tool to expand case detection. This could also address the problem of extensive under-diagnosis of HAT at PHC level, which has commonly been identified as a barrier to passive case-detection [18], [19], [20], [21]. A predictable drawback of such algorithms, however, is their low positive predictive value (PPV): HAT prevalence is typically low (<1–2% in endemic area populations of the most HAT-affected countries; 2.6%–10.8% in treatment-seeking populations presenting for PS [6]), meaning very high specificity is required to avoid massive over referral. This is difficult to achieve with high sensitivity.

Two studies [22], [23] have investigated the potential for HCWs to recognize and refer potential cases of HAT based on presenting symptoms. Boatin et al. (1986) identified a diagnostic scoring algorithm estimated to be 88% sensitive and 82% specific based on a comparison of mostly second stage, passively-detected *rhodesiense* HAT cases (which feature a different clinical profile and evolution than in *gambiense*) and both symptomatic and non-symptomatic controls in Zambia [23]. Jannin et al. (1993) performed a similar evaluation for *gambiense* HAT populations presenting for AS in the Republic of Congo (RC) and identified an algorithm that was 80% sensitive with a 20% PPV to detect parasitologically-confirmed cases [22].

A third study [24] describes an algorithm instituted in peripheral facilities in Democratic Republic of Congo (DRC) to determine the need for laboratory screening, but presents no information on its accuracy. An additional four studies have investigated the use of signs and symptoms for classifying disease stage and/or predicting a fatal prognosis among patients already confirmed as *gambiense* HAT cases; none identified a scoring system sufficiently specific to replace CSF white blood cell counting as a guide to therapeutic decision-making ([25], [26], [27] and secondary analysis of data presented in [28] by [29]).

We therefore evaluated the predictive value of these and new algorithms in a symptomatic, PS service-using population in the Nimule HAT focus of South Sudan to explore whether any would be appropriate for application in treatment-seeking populations at peripheral health facilities where laboratory testing is unfeasible. The algorithms were designed to be simple to assist HCWs with limited training. The performance of algorithms was also compared to referral decisions made by a panel of clinicians with extensive experience of diagnosing HAT in Africa.

## Methods

### Ethics statement

This study was approved by the London School of Hygiene & Tropical Medicine's ethical review committee and the Ministry of Health, Government (now Republic) of South Sudan. All participants provided verbal informed consent. Verbal consent was approved for use since most patients presenting to the service were not literate; receipt of consent was documented by laboratory staff on data collection forms.

### Study setting

HAT testing and treatment services in the Nimule focus of Magwi County, Eastern Equatoria State are available at a single site, Nimule Hospital, supported by the non-governmental organisation, Merlin (Medical Emergency Relief International) [13]. Services in this historic focus were re-introduced at the end of the Sudanese civil war, in 2005. Transmission is thought to have increased in recent years due to population movements of IDPs and returnees from neighbouring endemic areas. Small-scale AS surveys conducted in 2005, 2006 and 2008 revealed an estimated HAT prevalence of around 1% with the highest prevalence in any village estimated at 5.8% (Merlin programme data, unpublished).

Community health workers who have received nine months of formal clinical education are the most common cadre (46%) of staff involved in patient diagnosis and management in formal health facilities outside the hospital in the county, followed by staff with no formal clinical education (39%, mainly nursing assistants trained on the job), while only 15% are nurses and clinical officers [30].

### Collection of patient symptom data

Data on a list of 32 signs, symptoms and epidemiological criteria (henceforth collectively referred to as ‘symptoms’) were systematically collected from all patients tested for HAT at Nimule hospital over a seven month period from October 2009 to April 2010 (see Table 1). The list of symptoms included those used in other attempts to create HAT referral algorithms, clinical variables routinely collected at initiation of HAT treatment in the hospital and factors identified as important in local understandings of HAT from previous qualitative work in the study site [30]. Cases were defined as (i) positive microscopy on lymph fluid directly or on blood using capillary tube centrifugation (Woo test) or (ii) positive CATT on diluted blood serum at dilution 1∶16. Patients positive at dilution 1∶8 were considered serological suspects and followed-up after 3 months. During the study period, first line treatment was pentamidine for stage 1 and eflornithine for stage 2 cases.

**Table 1:** Presenting symptom data collected and used in algorithm construction.

Individual symptom	Method of ascertainment	Representation in algorithms after item reduction
Headache ≥1 week	Patient report	Retained
Back, neck or joint pain	Patient report	Body pains
Muscle pain	Patient report	Body pains
Fever ≥1 week	Patient report	Retained
Itchy skin	Patient report	Retained
Swollen face, legs or arms	Patient report	Oedema
Weight loss	Patient report	Retained
Generally poor state of health	Observation	Discarded
Decrease in appetite	Patient report	Appetite changes
Increase in appetite	Patient report	Appetite changes
Impotence	Patient report	Discarded
No menstruation	Patient report	Discarded
Enlarged lymph nodes	Examination	Cervical adenopathy
Insomnia	Patient report	Sleep pattern changes
Daytime sleeping	Patient report	Sleep pattern changes
Confusion or forgetfulness	Patient report	Abnormal behaviour
Aggressiveness	Patient report	Abnormal behaviour
Inactivity	Patient report	Abnormal behaviour
Hallucinations	Patient report	Abnormal behaviour
Convulsions	Patient report	Neurological problems
Difficult speaking	Observation	Neurological problems
Difficulty walking	Observation	Neurological problems
Patient unsteady	Observation	Neurological problems
Jerking movements	Observation	Neurological problems
Tremor in hands or lips	Observation	Neurological problems
Partial paralysis	Patient report	Neurological problems
Painful tibia/shin	Examination	Neurological problems
Treated for malaria/typhoid in last 2 weeks	Patient report	Recent malaria and/or typhoid treatment
Works/lives with cows	Patient report	Patient lives or works with livestock
Works/lives with goats	Patient report	Patient lives or works with livestock
Works/lives with sheep	Patient report	Patient lives or works with livestock
Works/lives with pigs	Patient report	Patient lives or works with livestock

All patient data were collected by laboratory staff (technicians and assistants without formal clinical training) before HAT test results were known. Lab staffs were trained to interview patients and recognise symptoms by the HAT programme manager (ES) and study coordinator (JP) before data collection. Interviews were private and patients could be tested without volunteering symptom information. Symptomatic, HAT-negative patients were advised to visit the hospital outpatient department for further management. Patients were only included in the analysis if they presented with at least one of the symptoms on the list and if complete lab test outcome data were available. Both naive and previously-treated patients were included.

### Estimation of algorithm performance

The 32 individual candidate symptoms were reduced to a more computationally and practically manageable set of 13 as follows. Odds ratios (ORs) were calculated for individual symptoms and for groups of similar symptoms (e.g., ‘sleep pattern changes’ encompassing ‘daytime sleeping’ and ‘insomnia’). Symptoms that were significantly associated with being a case or non-case only in individual analysis were not grouped with others. Symptoms that were infrequent in the data and/or showed no statistically significant association with case status were discarded (Table 1 and Table 2). R software, version 2.12 [31], was used to create all permutations of 4, 3, 2 and 1-symptom algorithms possible from the 13 symptoms shortlisted. Pragmatically, we considered that any algorithm consisting of more than four symptoms would be inappropriate for peripheral health facilities staffed by HCWs with minimal training. Algorithms were interpreted as indicating referral for patients presenting with any (as opposed to all) of the symptoms.

**Table 2:** Crude associations between the 13 symptoms used in algorithm construction and a positive HAT test.

Symptom	Cases (%) n = 27	Non-cases (%) n = 435	OR	95% CI	p-value
Body pains	85.2	83.5	1.3	0.4–4.5	0.650
Sleep pattern changes	66.7	54.9	1.6	0.7–3.7	0.238
Headache ≥1 week	55.6	59.3	0.9	0.4–1.9	0.700
Abnormal behaviour*	48.2	26.9	2.5	1.1–5.6	0.021
Fever ≥1 week	44.4	32.6	1.7	0.8–3.6	0.211
Itchy skin*	29.6	9.7	3.9	1.6–9.7	0.002
Appetite changes	22.2	14.7	1.7	0.6–4.3	0.295
Neurological problems*	22.2	4.8	5.6	2.5–12.4	<0.001
Oedema	14.8	5.3	3.1	1.0–9.8	0.051
Recent malaria or typhoid treatment	14.8	17.5	0.8	0.3–2.4	0.724
Weight loss*	11.1	1.8	6.7	1.6–27.2	0.007
Patient lives/works with livestock	11.1	10.3	1.1	0.3–3.7	0.899
Cervical adenopathy	3.7	3.0	1.2	0.2–9.9	0.834

* Significantly associated with being identified as a case, at p<0.05. Kerendel's sign (painful tibia) was present in 33.3% of cases and independently significantly associated with a positive test outcome (individual OR 5.9, p-value <0.001) but was combined with other more rare symptoms into the larger category ‘neurological problems’. There were no significant differences in demographic characteristics (age, sex, residency status, location) between cases and non-cases (data not shown). OR: Odds ratio. CI: Confidence interval.

Each algorithm was then tested against the patient data to compute sensitivity, specificity, PPV and NPV of each, assuming results of lab testing to be a gold standard diagnosis of HAT. The Boatin et al. (1986) and Pepin et al. (1989) algorithms were also tested on the data, as well as three algorithms from the Jannin et al. (1993) study: the best performing algorithm for detecting all HAT patients, including those diagnosed on serology grounds alone (‘Jannin-all’ algorithm) and the two best-performing algorithms for detecting parasitologically confirmed cases, which in the original study setting yielded a sensitivity of 84% with a PPV of 9% (‘Jannin-para2’ algorithm) and 80% sensitivity, 20% PPV (‘Jannin-para3’ algorithm). Minor deviations from the algorithms as originally published were made due to the way data were collected in this study (Table 3).

**Table 3:** Modified algorithms from other HAT studies tested using Nimule Hospital data.

	Algorithm				
Symptom	Boatin	Pepin	Jannin-all	Jannin-para2	Jannin-para3
Headache ≥1 week	2	1	1	1	1
Fever ≥1 week	2	-	1	1.5	1.5
Fever unresponsive to anti-malarial	-	1	-	-	-
Oedema	2	-	3	3	3
Itching	2	1	-	1	1
General body pain	1	1	-	-	-
Weight loss	-	1	-	-	-
Sleep problems	1	1	0.5	-	-
Neurological problems	-	-	0.5	-	-
Cervical adenopathy	-	-	4.5	4.5	4.5
Livestock in compound	-	-	1	1	1
(Family history of HAT)*	-	-	1	1.5	1.5
**Threshold score for referral**	**≥4**	**≥1**	**≥1**	**≥2**	**≥3**

Numbers indicate scores attributed to each symptom, if present.

* Data on this symptom were not collected in this study.

### Comparison with expert clinician performance

Four clinicians with expertise in HAT patient management were asked to review anonymised patient symptom data and, blinded to the test outcome, decide whether they would have referred the patient for HAT testing. These experts (ES, AL, AP, SK) were selected for their substantial experience in direct HAT patient management in *T.b. gambiense*-endemic areas (South Sudan, DRC, RC and Central African Republic). Experts were told only that patients came from an area with about 1% HAT prevalence and were asked to make referral decisions (yes or no responses allowed only) as if they were working in a PHC facility without HAT testing capacity, one day's walk from the HAT treatment centre. The only other information provided on patients was sex, age (≤14 years or >14 years) and whether the patient had ever been treated for HAT. Accuracy indicators were computed as above. Qualitative comments from experts about some of the difficulties they encountered in assigning referral decisions were taken into account when interpreting subsequent analyses.

The extent of agreement among experts' referral decisions was computed so as to establish the level of consensus about what an appropriate clinical picture for HAT referral might be. Cohen's kappa was used to measure inter-rater agreement between pairs of raters and Fleiss' kappa between multiple raters [32]. Cases for which experts unanimously agreed to refer were explored to identify symptoms that were strongly associated with a unanimous decision to refer. So as to account for potential confounding in these associations, a generalised linear model with robust error variances [33] was used to estimate these associations by including all symptoms significant at the 90% confidence level in univariable analysis into a multivariable model, using a forward stepwise procedure. The final model contained all symptoms that remained significantly associated at the 95% level. Age, sex and previous HAT treatment history were considered as potential confounders for referral, and were forced into the model to adjust for their potential effect. All statistical analyses were performed using Stata software, version 11 (StataCorp, Texas 2009).

## Results

### Performance of syndromic algorithms

Complete symptom and test outcome data were available for 462/652 (70.9%) patients tested passively for HAT during the 7 month study period. Incomplete symptom data were largely due to a staff shortage in the hospital laboratory over a 10 week period. 27/462 patients were confirmed as cases, of whom 24 (89%) were parasitologically confirmed and 24 (89%) were in stage 2, yielding a prevalence of 5.8% among patients tested.

**Figure 1:**
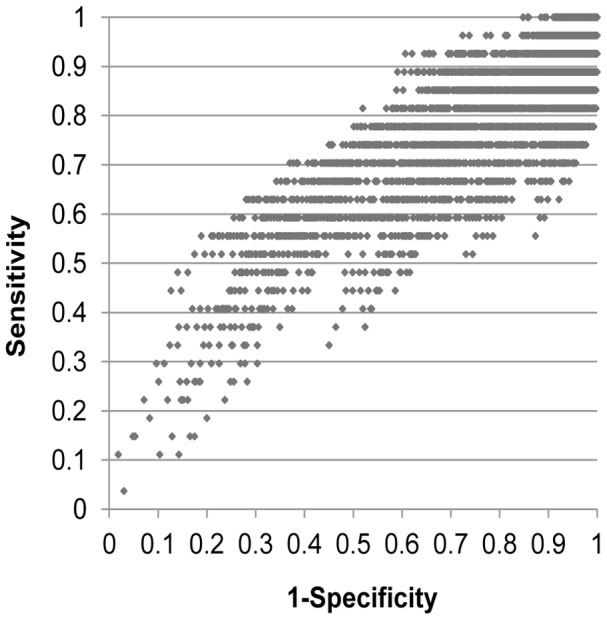
Receiver operating curve diagram of all candidate syndromic algorithms evaluated. Each point represents the sensitivity and 1-specificity of a single algorithm. Ideally, the highest performing algorithms would be located in the top left corner of the graph.

Out of 14,067 possible candidate algorithms evaluated, more than half showed a sensitivity greater than 95%, but specificity and PPV were uniformly low, and the latter remained <15% even for algorithms that had a sensitivity as low as 50% (median sensitivity 96.3% (inter-quartile range: 88.9–100.0%), specificity 1.6% (0.2–6.7%), PPV 5.8% (5.7–5.9%) and NPV 94.9% (87.5–100.0%), Figure 1). The best-performing algorithms consisted of three core symptoms (sleep problems, neurological problems and weight loss), with or without a history of oedema, cervical adenopathy or proximity to livestock (algorithms 4, 6, 8 and 10 in Table 4, sensitivity 88.9–92.6%, PPV 8.4–8.7). Algorithm 4 (sleep problems AND/OR neurological problems AND/OR weight loss AND/OR oedema) appeared to offer the highest combination of sensitivity (92.6%) and PPV (8.7%).

**Table 4:** Candidate syndromic algorithms with the 10 highest PPVs and sensitivity ≥75%.

Algorithm	Sx n	Body pains	Sleep pattern change	Headache ≥1 week	Abnormal behaviour	Fever ≥1 week	Neurological problems	Itchy skin	Appetite changes	Oedema	Malaria/typhoid	Weight loss	Livestock	Cervical adenopathy	Patients referred (%)	Sensitivity (%)	Specificity (%)	PPV (%)	NPV (%)
1	4				Y	Y				Y				Y	248 (53.7)	81.5	48.0	8.9	97.7
2	4				Y		Y		Y		Y				239 (51.7)	77.8	49.9	8.8	97.3
3	4					Y	Y	Y					Y		242 (52.4)	77.8	49.2	8.7	97.3
4	4		Y				Y			Y		Y			289 (62.6)	92.6	39.3	8.7	98.8
5	3				Y	Y				Y					245 (53.0)	77.8	48.5	8.6	97.2
6	3		Y				Y					Y			281 (60.8)	88.9	40.9	8.5	98.3
7	4				Y	Y				Y		Y			246 (53.2)	77.8	48.3	8.5	97.2
8	4		Y				Y					Y	Y		295 (63.9)	92.6	37.9	8.5	98.8
9	4			N				Y					Y	Y	249 (53.9)	77.8	47.6	8.4	97.2
10	4		Y				Y					Y		Y	287 (62.1)	88.9	39.5	8.4	98.3

Sx n: The number of symptoms in the algorithm. Y/N: According to the algorithm, the symptom should (Y/yes) or should not (N/no) be present in the patient being referred. When used by HCWs in practice, algorithms containing more than one symptom should be read as indicating referral if patients present with any (as opposed to all) of the symptoms listed, i.e., symptom A and/or symptom B and/or symptom C and/or symptom D. Patients referred = the number of patients out of 462 who matched the algorithm being tested.

The number of presenting HAT symptoms did not appear to predict infection well: algorithms based on the number of presenting HAT symptoms (regardless of which) fared worse at predicting HAT infection than the best-performing algorithm identified above (e.g. the presence of ≥4/13 symptoms, regardless of which, yielded a sensitivity 63% and PPV 8.5; other data not shown).

The Boatin, Pepin and Jannin algorithms also featured low PPV and, in addition, for the Boatin and Jannin algorithms, low sensitivity; the Pepin algorithm featured very high sensitivity but very low specificity (Table 5).

**Table 5:** Performance of previously published syndromic algorithms.

Algorithm	Patients referred (%)	Sensitivity %	Specificity %	PPV %	NPV %
Boatin	244 (52.8)	70.4	48.3	7.8	96.3
Pepin	455 (98.5)	96.3	1.4	5.7	85.7
Jannin-all	362 (78.4)	85.2	22.1	6.4	96.0
Jannin-para2	163 (35.3)	55.6	66.0	9.2	96.0
Jannin-para3	65 (14.1)	29.6	86.9	12.3	95.2

### Performance of expert clinicians

When referral decisions were compared to test outcome data, expert referrers consistently would have referred more cases than non-cases for screening (Table 6). However, although 3/4 referrers had high sensitivity, their PPVs were lower than the best-performing algorithm identified in this study, corresponding to a higher proportion of patients referred overall (≥75% of patients, as compared to 60%). 62.2% (255/410) of patients were unanimously referred while only 7.1% of patients in the data would not have been referred by any expert.

**Table 6:** Performance of expert referrers.

	Cases referred (%)	Non-cases referred (%)	Chi^2^ p-value	Total pts referred (%)	Sens %	Spec %	PPV %	NPV %
**Referrer 1**	20 (74.1)	248 (57.0)	0.081	268 (58.0)	74.1	43.0	7.5	96.4
**Referrer 2**	26 (96.3)	356 (81.8)	0.054	382 (82.7)	96.3	18.2	6.8	98.8
**Referrer 3**	26 (96.3)	321 (73.8)	0.009	347 (75.1)	96.3	26.2	7.5	99.1
**Referrer 4** *	26 (100.0)	345 (89.8)	0.088	371 (80.3)	96.3	89.0	7.0	100.0

* No decision for 52 patients. Pts: patients. Sens: sensitivity. Spec: specificity.

### Expert approaches to syndromic HAT referral decisions

Good agreement on expert referral decisions, with an overall kappa score of 0.56, suggested that experts broadly agreed on what *should* constitute an ‘appropriate’ HAT referral, however, agreement on referral of actual cases was in fact quite poor (0.27 for cases, 0.57 for non-cases) (Table 7) [34]. Multivariable analysis of symptoms associated with unanimous referral provided insight as to what experts considered appropriate conditions for referral. Five symptoms (sleep pattern changes, cervical adenopathy, neurological problems, recent malaria/typhoid treatment and abnormal behaviour) retained significance in the final multivariable model at 95% confidence, after adjustment (Table 8). The only single-symptom algorithm associated with unanimous referral was ‘sleep pattern changes’. A combination of previous HAT treatment history and any HAT symptom led to automatic referral for only one expert referrer. Adults (65.5%) were significantly more likely to be referred than children under 15 years (43.1%) (p-value 0.002). There was generally better agreement about what constituted an appropriate referral between expert referrers and the Boatin algorithm than with the Pepin or Jannin algorithms.

**Table 7:** Kappa scores assessing agreement between all pairs of expert referrers and expert referrers with algorithms from other HAT studies.

	Ref 2	Ref 3	Ref 4	Boatin	Pepin	Jannin-all	Jannin-para2	Jannin-para3
**Ref 1**	0.42	0.53	0.30	0.33	0.03	0.09	0.10	0.10
**Ref 2**	-	0.77	0.85	0.38	0.07	0.17	0.19	0.07
**Ref 3**	-	-	0.59	0.31	0.04	0.13	0.12	0.07
**Ref 4**	-	-	-	0.24	0.03	0.24	0.10	0.03

Ref: Referrer. Kappa scores range from 1 representing complete agreement to −1 representing complete disagreement; a score of 0 represents no more agreement than would be expected due to chance.

**Table 8:** Multivariable model of key HAT symptoms associated with unanimous expert referral, adjusted for age, sex and previous HAT treatment history (n = 407).

Variable	RR-adjusted	95% CI	p-value
**Potential confounding variables**			
Adult age	1.8	1.4–2.4	<0.001
Male sex	1.0	0.9–1.1	0.703
Patient treated for HAT before	0.8	0.5–1.2	0.284
**HAT-specific symptoms** *			
Sleep pattern change	2.9	2.3–3.7	<0.001
Cervical adenopathy	1.8	1.2–2.5	0.003
Neurological problems	1.4	1.3–1.7	<0.001
Malaria/typhoid treatment	1.3	1.2–1.5	<0.001
Abnormal behaviour	1.2	1.1–1.4	0.001

* An additional symptom, body pains, was moderately significant in the final model (p-value 0.051).

## Discussion

### Using algorithms to identify HAT syndromically

This study suggests that, for users of a PS service in this South Sudanese focus, algorithms developed for syndromic diagnosis of HAT in other settings have low sensitivity and poor PPV. Ours is the first study specifically to examine the potential effectiveness of syndromic algorithms in a treatment-seeking population and we present here, a simple four-item syndromic algorithm (sleep problems AND/OR neurological problems AND/OR weight loss AND/OR a history of oedema) which had good sensitivity (92.6%) for detecting HAT in such a context. Under this algorithm, and given the prevalence observed in Nimule among patients presenting for testing, about 9 out of 10 referred patients would be non-cases, corresponding to a relatively low PPV. This PPV may, however, be considered an acceptable harm for both patients and health services given the benefit of detecting what is an almost universally fatal disease. Indeed, such an algorithm may allow peripheral HCWs to supplement existing passive and (intermittent) active case finding, and promote integration and strengthening of HAT services within the overall health system. As with all algorithms derived from a single patient group it will need to be tested in independent populations before being recommended for clinical practice, but we consider it is simple enough to be usable by HCWs with limited training.

Expert opinion on the potential of syndromic detection of HAT appears to be unreconciled in the academic literature. Descriptions of how to recognise a case of HAT syndromically abound in the historic and contemporary HAT literature [3], [27], [35], [36], [37], [38], [39], [40], [41], [42] and the presence of one particular sign, cervical adenopathy, was the most important pre-condition for laboratory testing in AS campaigns over most of the 20th Century. On the other hand, formal guidance discusses the challenge of operationalising existing syndromic detection techniques, preferring instead to advocate widespread use of the more sensitive diagnostic technologies developed and refined over the last three decades to avoid the risk of under-detection of a fatal disease [5], [6], [43]. There has been much less discussion of the risk of under-detection of patients who cannot access these technologies directly when, because of operational realities, use of these technologies is not widespread, and who might otherwise benefit from a syndromically-based referral. Perhaps as a result, there are no guidelines targeted to HCWs working in peripheral facilities to help them identify suspect patients in these instances.

Other authors have accepted an algorithm with high sensitivity but low PPV where diagnostic facilities were easily at hand, as in Pepin et al.'s (1989) study, which equipped all peripheral health facilities in the district with microscopes; this would however have greatly increased these HCWs' workload and in the current South Sudan context seems unrealistic. Boatin et al. (1986) cautiously proposed the utility of algorithms with high sensitivity but no PPV data for application in HCW referrals of *rhodesiense* HAT, which occurs sporadically over large areas and thus requires effective passive case detection. Jannin et al. (1993), however, rejected algorithms featuring <10% PPV (albeit with lower sensitivities) because of the heavy workload implications for HCWs. The burden placed on patients to travel to a central testing facility, most of whom would be found negative, was also considered too great by these authors. However, in our study setting of poorly accessible PS services and nonexistent AS, an algorithm with 10% PPV would avert approximately one death for every ten already treatment-seeking patients who were referred to the testing centre. Our panel of experts also appeared to implicitly accept this high rate of false positives, given that they chose to refer a majority of patients in the case series despite knowing that HAT prevalence was 1% in the study area.

The reduced accuracy in Nimule of syndromic algorithms developed in other settings may be due to the lower proportion of symptomatic patients in those cohorts, as already discussed. It may also, however, be due to cultural and linguistic differences in patient perceptions and descriptions of disease. For this reason, it would be useful to validate the algorithm more rigorously in an independent group of patients, first in Nimule and then, if accuracy is confirmed, elsewhere, to explore its applicability in a range of health service contexts. In a high HIV burden setting, three of the four symptoms in this algorithm (neurological problems, weight loss and oedema) could signal various opportunistic infections associated with AIDS, thus further reducing the algorithm's specificity; on the other hand, these patients would also benefit from hospital referral.

Out of the four symptoms, ‘sleep problems’ was the most frequently reported in cases and thus made the greatest contribution to overall sensitivity; however, excessive sleeping (included in this symptom grouping) was frequently reported by both cases (63.0%) and non-cases (49.4%), suggesting that HCWs may face difficulties reliably evaluating its presence in patients. By contrast, neurological symptoms, weight loss and oedema were more discriminating for infection, and yet were rarely associated with HAT by peripheral HCWs interviewed as part of additional research in Nimule (to be published separately) [30]. Weight loss and oedema were also not strongly associated with unanimous expert referral after accounting for confounding, suggesting that to experts, too, these may be counter-intuitive referral criteria. Although there appeared to be consensus among them on what should constitute an appropriate referral, there was poor agreement on referral decisions for true cases, suggesting that many of these true cases did not match what could be considered their ‘consensus case definition’. Alternatively, it may expose difficulties experts faced in making decisions about these symptoms with limited information due to the study design.

### Limitations

Five main limitations affect interpretation of this study's findings. First, patient symptom reporting may have been subject to respondent interview bias, including culturally-specific interpretations of some types of symptoms associated with HAT and other conditions. How patients report HAT symptoms in other areas may affect this algorithm's generalisability.

Second, data quality may have been affected by the skill of laboratory personnel responsible for collecting them. This has been recognised as a challenge in other studies of HAT symptomatology [44] and may be particularly problematic for recognition of cervical adenopathy [2], [22]. It is possible that some HAT symptoms more prevalent in true cases were under-detected and therefore had a lower probability of being selected in the final algorithm. It is debatable whether most referring HCWs in peripheral facilities would possess a higher level of clinical skill than the lab attendants trained in this study; if not, the symptoms in this final algorithm may reflect what is, in fact, most practical, in this context. Outside of a research setting, hospital lab staff would not be expected to be involved in syndromic screening since the more sensitive CATT-WB would be available.

Third, the ‘gold standard’ we used to identify cases in our analysis was the diagnostic algorithm used in the Merlin HAT programme, which, as any HAT diagnostic algorithm, is dependent on the performance of all tests within it and probably has a true sensitivity of between 85–90% and a PPV of around 90% in PS settings [6]. Little is known about the symptom profile of false negatives excluded by this diagnostic algorithm so it is difficult to predict what effect, if any, these exclusions would have on the sensitivity of the syndromic algorithms we present here. The Merlin diagnostic algorithm is also probably more sensitive than gold standard diagnostic algorithms used to evaluate the syndromic algorithms presented from older studies, making comparisons less straightforward.

Fourth, our algorithm findings are applicable mainly to detection of stage 2 patients, since so few patients in stage 1 were included in the case series. The sensitivity of our algorithms may be lower in routine peripheral settings if the typical clinical profile of HAT cases presenting there is less or differently symptomatic, with proportionately more patients in stage one seeking care.

Finally, our relatively small sample size of 27 cases limits the precision of our estimates of algorithm sensitivity and PPV. Consideration of their 95% confidence intervals (e.g., 75.7–99.1% for sensitivity and 5.7–12.5% for PPV in Algorithm 4) suggests that they may be considered reasonably accurate but the sample size may affect the precise ranking of algorithms; practitioners could, for example, choose to implement or validate any of the top-performing algorithms identified in this study (algorithms 4, 6, 8 and 10 in Table 4) according to the feasibility of teaching and using them.

### Conclusions

Current HAT diagnostic algorithms are too complex for use in peripheral health structures, and there is very little guidance available to HCWs working in these areas on when to consider referring suspected patients to a central testing facility based on symptoms. This is especially problematic in a context with low AS coverage like the Nimule focus where most HAT patients in the periphery are not detected, or in outbreak situations where funding and capacity for AS is often initially unavailable.

The simple four-symptom referral algorithm identified in this study has the potential to avert one death through testing and treatment for every ten patients referred and to identify most symptomatic HAT patients, if systematically applied. If our findings can be validated in an independent sample, this algorithm could represent a useful additional tool for control programmes to improve case detection in the periphery and promote integration of HAT services within overall health systems at reasonably low added cost.
